# The Flow of Information in Trading: An Entropy Approach to Market Regimes

**DOI:** 10.3390/e22091064

**Published:** 2020-09-22

**Authors:** Anqi Liu, Jing Chen, Steve Y. Yang, Alan G. Hawkes

**Affiliations:** 1 School of Mathematics, Cardiff University, Cardiff CF24 4AG, UK; ChenJ60@cardiff.ac.uk; 2 School of Business, Stevens Institute of Technology, Hoboken, NJ 03070, USA; steve.yang@stevens.edu; 3 School of Management, Swansea University, Swansea SA1 8EN, UK; a.g.hawkes@swansea.ac.uk

**Keywords:** information entropy, market information flows, trading behavior identification, news sentiment

## Abstract

In this study, we use entropy-based measures to identify different types of trading behaviors. We detect the return-driven trading using the conditional block entropy that dynamically reflects the “self-causality” of market return flows. Then we use the transfer entropy to identify the news-driven trading activity that is revealed by the information flows from news sentiment to market returns. We argue that when certain trading behavior becomes dominant or jointly dominant, the market will form a specific regime, namely return-, news- or mixed regime. Based on 11 years of news and market data, we find that the evolution of financial market regimes in terms of adaptive trading activities over the 2008 liquidity and euro-zone debt crises can be explicitly explained by the information flows. The proposed method can be expanded to make “causal” inferences on other types of economic phenomena.

## 1. Introduction

The financial market is a natural arena for information competition and investors often seek to collect and process information to assist their investment decisions marking [1,2]. With the proliferation of the electronic trading, the quality and timeliness of information become, in particular, highly important for traders. Investigating how traders use information become vital to comprehensively analyze and understand important finance problems including price formation, price discovery and market efficiency [3,4,5,6,7,8]. Often, new financial technologies offer greater capacity to process larger amount information more efficiently that would result in faster price discovery [9,10,11] and eventually more efficient market as the Efficient Market Hypothesis (EMH) states. However, the EMH only presents novelty of a basic classification of information used in the financial market. New types of information such as business news that popularized through the information technology revolution are not considered. Moreover, the advancement of financial technologies has implicitly increased the complexity of the market; thus, how the multiple information transmits and influences one another through trading decisions is much more complex and has exceeded what the EMH can describe. Therefore, we endeavor to propose a new method based on entropy to identify the roles of different information sources in price formation within the context of a contemporary financial market.

Entropy, by definition, is proposed to calculate the amount of information contained in a signal series. This concept is also associated with the second law of thermodynamics and is used to calculate the change of states of a system. The modern financial market clearly forms a natural new venue to apply such method. Up to date, there have not been many studies applying entropy to finance problems comprehensively. Reference [12] detected significant information transition between the Dow Jones and the DAX indexes and [13] calculated transfer entropy of the VIX and the iTraxx Europe index to examine relative power of market risk and credit risk. Reference [14] used Rényi’s information flow to conduct similar experiments on S&P500 and DAX indexes. Reference [15] expanded the analysis to of information flows of market volatility. More recent studies [16,17] brought more insights of information flows in commodity markets. However, all these studies focused on analysis of financial time series and statistical interpretations of financial data. To our best knowledge, there have been no studies using entropy to describe the complex financial system based on multivariate information flows; nor further identifying trading activities that are driven by various types of information.

The contemporary financial market is primarily based on electronic trading, and both real-time market data and business news are two dominate types of information that feed into trading decisions. Traders are forced to discover more information to compete with others, especially when profitability of traditional trading rules (e.g., technical analysis) are reduced in the so-called “zero-sum game”. Furthermore, textualization techniques have developed rapidly and it becomes a general practice that professional traders track social media messages and business news (Humphries, Lewis (3 February 2012). “The Power Of Social Media: Influencing Trading And The Markets.”). Reference [18] suggests that many institutional investors and high frequency traders have adopted news feeds to generate investment signals and determine trading timing. Several academic research studied the relations between news sentiment and stock markets (see [19,20,21]). However, there has been no study examining such relations through information flows. This is vital as we have emphasized earlier that price formation and market efficiency are essentially driven by information transmission; thereby, to understand how information flowing within the financial system is the key to answer these questions. Furthermore, in the complex system, information flows would interact with one another, which forms dynamic mechanisms among different market conditions in relation to the news and traditional real-time market data (e.g., returns).

**Figure 1:**
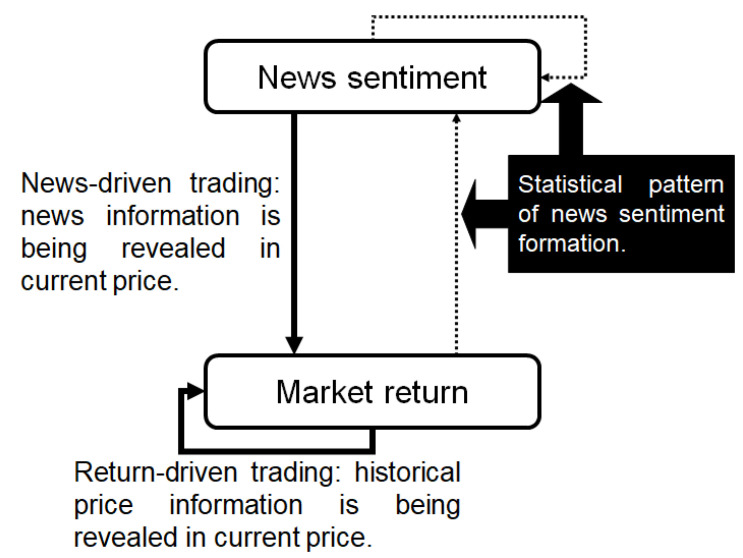
Information flow diagram.

In Figure 1, we model the financial market as a bi-variate system, in which news sentiment and market returns are two types of information that guide trading decisions and there are flows within and between them. Entropy, as a way of describing the dynamic feature of the system, will be introduced to quantify the information flows. Technically, we measure two information flows: one is the flow in the underlying process itself and the other is from the news sentiment to price movements; and these two flows indicate return-driven and sentiment-driven trading respectively.

Finance literature typically turns to causality analysis to understand the information transmissions among data series. However, to model a system involving multiple series, the simple uni- or bi-directional causal relationships become insufficient to describe the mechanism that possibly works more like a dynamic network. Entropy is an expression of randomness or lack of information of a system. It involves dynamic and non-symmetric measures (e.g., transfer entropy) that are able to reveal statistical relationships regardless of data linearity and normality. Regarding this, entropy has been applied to social networks [22], information transmission across financial assets [23,24,25], causal influences and applied statistics [26,27,28], and in dynamic systems [29,30]. As mentioned, a few studies have applied the transfer entropy to justify the coupling between two financial time series (see [12,13]). In addition to the advantage in capturing non-linear relationships, the entropy method treats information in a way that is close to how traders make trading decisions in reality. In contrast to the standard models (e.g., VAR, Granger causality test) that present impacts of lagged data separately, entropy takes information filtration to indicate the use of all useful information up-to-date. Such a method apparently presents a better way to approximate real trading behavior. Indeed, we have constructed an entropy-based modeling framework, as an alternative method to classic modeling techniques, to describe the multiple information flows in the financial market in [31]. In this study, we extend this previous work to develop a method to identify trading behaviors based on information sources. We believe the entropy-based information flows would allow us to quantify the impact of the various information flow, hence, accurately categorize return-driven and sentiment-driven trading.

We evaluate conditional entropy and transfer entropy to accommodate different trading activities in the complex market structure and model the information flows within and between different types of information sources (see Figure 1). We use the Thomson Reuters News Analytics database to compute news sentiment and to enrich interpretations of news-driven trading activities. The Standard & Poor’s 500 index (.SPX) is applied to identify the return-driven trading activities. These results allow us to clearly distinguish two different trading behaviour. Over time, when a particular trading pattern persists, the market may experience a regime change that could potentially contribute to the literature on market regime and structure studies as it provides a way potentially quantifies the the efficiency change of the market. The normal market conditions could be driven by return-driven trading as the EMH normally hypothesized, or a mixture of return- and sentiment-driven trading that may constantly reinforce each other. However, when the market experiences unusual conditions such as financial crises, we observe that such patterns are disrupted. In particular, return-driven activities lose their persistence manifested in the sharp drop of information flow. Instead, the sentiment-driven trading becomes dominant. This means what determines the trading decisions is associated with investors’ “needs” from the market. For example, after the bubble bursts, most investors sense fear of crisis and their “needs” would shift from making profits to escaping losses. Not only we provide consistent arguments with some early research such as [32,33,34], we bring contributions to an important part of literature here: the financial market would always have a certain level of self-adjustment and self-recovery ability in response to information shocks. However, once the scale of the return or sentiment driven trading activities turn overwhelmingly dominant and exceed a certain boundary and/or threshold, the market may move towards structural changes. This would bring new insights to studies on market regime shifts.

To sum up, we consider the financial market as a bi-variate system composed of two types of information: market returns and news sentiment (see Figure 1). We use information flows measured by entropy in this system to identify trading behaviors and the potential impact of concentrated activities in one of these trading to move the market. The rest of the paper is structured as follows. Section 2 interprets the entropy measures that are adopted to evaluate information flow and the methodologies to formulate different types of trading activities and market regimes. Section 3 summarizes the news and market data. Section 4 presents results of trading and market regime identification. Finally the paper concludes in Section 5 by assessing results, contributions and limitations.

## 2. Methodology

In this section, we outline the entropy-based method to evaluate information flows in the financial system in order to detect market-, news- or mixed-driven trading activities. The rationale of this trading behavior identification method is that investors not only adopt but also “generate” information through their trading and these market-wide trading activities will be translated into the information transmission process and eventually reflected in price movements. Hence, information flows in the system reveals the type of information applied by investors into their trading decisions. Considering the most widely adopted information sources, the information flow from market returns to returns indicates return-driven trading while that from news sentiment to market returns indicates news-driven trading. These two types of trading can coexist, which coincides with a mixed impact to market and we call it mixed-driven trading. To further characterize the overall market situation, we establish information-based market regimes that are linked with trading behaviors, namely the return-driven, the news-driven, and the mixed (both return and news) regimes to demonstrate the market-level shifts that caused by dominant impacts from these trading activities.

### 2.1. Entropy, Information Flows and Trading

The financial market that evolves through information transmission can be framed into a bi-variate system with two information sources: market returns and news sentiment. To start, we define notations in the financial market model. The market return series is denoted by $R=\{r_{1},r_{2},r_{3},\ldots \}$ and the news sentiment series is denoted by $S=\{s_{1},s_{2},s_{3},\ldots \}$. These two types of information can form four information flows transmission (see Figure 1) that have been well explored in our previous work [31]. To directly observe and classify trading behaviors, we only need to consider the information flows that ultimately reflect price movements, namely: (1) market returns → market returns ($I_{R\to R}$); (2) news sentiment → market returns ($I_{S\to R}$). Market returns and news sentiment are sources of information transmit in $I_{R\to R}$ and $I_{S\to R}$ respectively, ultimately drive the underlying price process to evolve. From the perspective of traders, they often analyze market data and news and respond to them directly to make investments. Aggregation of the these decision making activities drives market prices to fluctuate or the entire market condition to shift (e.g., herding behaviour). Therefore, if we identify information flows targeting the changes of the market returns, we can find out what causes the market movements, which is consistent with the theory of price discovery. We establish information entropy as a measure to demonstrate complex causality relationships in the financial system. We describe the relation between an information flow (e.g., $I_{R\to R}$) as “self-causality” (see [35]) and the relation between two different information flows (e.g., $I_{S\to R}$) as “cross-causality”. We present how to quantify them using conditional entropy and transfer entropy respectively in the following sections.

#### 2.1.1. Entropy Measures

If the event space *X* is a time series, it involves a special case of joint probability space—the observations of sub-series. If we denote *k* as the number of consecutive observations until time *t* as $x_{t}^{\left(k\right)}=x_{t},x_{t-1},\ldots \ldots ,x_{t-k+1}$, the entropy of $x_{t+1}$ that is conditioned on previous observations $x_{t}^{\left(k\right)}$ can be written as Equation (1).

(1) $$\begin{array}{cc} h_{X}\left(k\right) & =H_{X}\left(x_{t+1},x_{t}^{\left(k\right)}\right)-H_{X}\left(x_{t}^{\left(k\right)}\right)\\ \; & =-\sum p\left(x_{t+1},x_{t}^{\left(k\right)}\right)\log_{2}p\left(x_{t+1}|x_{t}^{\left(k\right)}\right) \end{array}$$

in which $H_{X}$ is the Shannon entropy defined as

$$H_{X}=-\sum p\left(x_{t}\right)\log_{2}p\left(x_{t}\right).$$

Please note that the summation in this equation is over all possible values of $\left(x_{t+1},x_{t}^{\left(k\right)}\right)$ for fixed *t*, but if the time series *X* is stationary, the result $h_{X}\left(k\right)$ will be independent of *t*. This is also called conditional block entropy, in which *k* is the block length. Increasing *k* will result in decreasing $h_{X}\left(k\right)$ as long as $x_{t-k}$ contains more information than $x_{t-k+1}$ to forecast $x_{t+1}$ [12]. Here, *k* can also be interpreted as the memory length of *X* if and only if $h_{X}\left(k\right)=h_{X}\left(k+1\right)$.

Schreiber [36] proposed the transfer entropy that quantifies asymmetric dynamics of two processes (Equation (2)). It denotes that, despite information collected from $x_{t}^{\left(k\right)}$, information in $y_{t}^{\left(l\right)}$ may also be valuable in the prediction of $x_{t+1}$. Obviously, $T_{Y\to X}\left(k,l\right)=0$ if $y_{t}^{\left(l\right)}$ has no additional influence on $x_{t+1}$ after subtracting information already involved in $x_{t}^{\left(k\right)}$.

(2) $$T_{Y\to X}\left(k,l\right)=\sum_{x,y}p\left(x_{t+1},x_{t}^{\left(k\right)},y_{t}^{\left(l\right)}\right)\log_{2}\frac{p(x_{t+1}|x_{t}^{\left(k\right)},y_{t}^{\left(l\right)})}{p(x_{t+1}|x_{t}^{\left(k\right)})}$$

Indeed, the transfer entropy can be formulated using conditional block entropy (see Equation (3)).

(3) $$\begin{array}{cc} T_{Y\to X}\left(k,l\right)= & \sum_{x,y}p\left(x_{t+1},x_{t}^{\left(k\right)},y_{t}^{\left(l\right)}\right)\log_{2}\frac{p(x_{t+1}|x_{t}^{\left(k\right)},y_{t}^{\left(l\right)})}{p(x_{t+1}|x_{t}^{\left(k\right)})}\\ = & \sum_{x,y}p\left(x_{t+1},x_{t}^{\left(k\right)},y_{t}^{\left(l\right)}\right)\log_{2}p(x_{t+1}|x_{t}^{\left(k\right)},y_{t}^{\left(l\right)})-\sum_{x}p\left(x_{t+1},x_{t}^{\left(k\right)}\right)\log_{2}p(x_{t+1}|x_{t}^{\left(k\right)})\\ = & h_{X}\left(k\right)-\left(H_{X,Y}\left(x_{t+1},x_{t}^{\left(k\right)},y_{t}^{l}\right)-H_{X,Y}\left(x_{t}^{\left(k\right)},y_{t}^{l}\right)\right)\\ = & h_{X}\left(k\right)-h_{X,Y}\left(k,l\right) \end{array}$$

in which the second term $h_{X,Y}\left(k,l\right)$ indicates the conditional entropy of *X* give the block information of both $x_{t}^{k}$ and $y_{t}^{l}$. This transformation suggests that the transfer entropy $T_{Y\to X}\left(k,l\right)$ evaluates the amount of information explained by $y_{t}^{l}$ when $x_{t}^{k}$ is already taken into account.

#### 2.1.2. Entropy as a Causality Measure

In finance studies, whether a factor produces significant impacts to markets is usually examined by the Granger causality test. However, the normality and linearity assumptions of this test can cause inaccurate results for financial data. For instance, when we consider price movements, they do not always nicely follow the random walk, instead, trends, reversal as well as seasonal patterns are often observed and used as analyst tools that are impossible to be well modeled linearly. Furthermore, when the financial system’s complexity increases as our bi-variate system indicates, the impacts of news to the market would be too complex to be captured by a linear model. Entropy measures, in contrast, will be able to offer better and more flexible ways to test and quantify impacts of a variety of information to price movements. In fact, when observations in a time series are independent, the entropy would not reduce by involving memory of previous observations; when two time series are independent from each other, the transfer entropy between them will be zero. Moreover, when the variables involved in the system are multivariate normal, the transfer entropy would be equivalent to the Granger causality test. The equality between the causality and entropy measures can be present the following three theorems and we provide proofs in the Appendix A (also see [31]).

**Theorem** **1.** *If X is a sequence of i.i.d. random variables, then there is no self information flow within the series X i.e., the conditional block entropy shall be equal to the Shannon entropy.*

**Theorem** **2.** *For two independent series X and Y, the transfer entropy between them will be zero (i.e., no causal relationships between X and Y).*

**Theorem** **3.** *Granger causality and transfer entropy are equivalent if all variables involved are distributed as multivariate normal distributions.*

Therefore, entropy measures, in theory, should not only provide consistent results with the classic methodologies for Gaussian variables that have linear relationships, but also accommodate non-normal and non-linear properties that standard methods would fail to identify. In addition, entropy measures enable the idea of capturing impacts of a block of information which is far better in describing the information processing behavior in real trading practice than the standard models (e.g., vector autoregression, Granger causality test) which presents impacts of different “lags” separately. These features will, inevitably, make entropy measures more suitable and robust for financial modeling of a complex market. We have provided the detailed comparison between the entropy and linear modeling of our bi-variate system that approximates the financial market in the Appendix B and the conclusion is that the linear models are less consistent and entropy approach provides additional insights, especially when dealing with a new type of financial data, such as ‘news sentiment’.

In the financial market, what would fundamentally move the prices is trading activities, i.e., price increases with rising buying power and vise versa. The entropy measures (see Section 2.1.1) quantify the changes of states given previous information. In our model, they can indicate traders’ responses to different information with subsequent price movements. To be specific, the conditional block entropy of the return series tells how traders responding to price information and the transfer entropy from news to returns explains how traders reacting to news information. In this way, the information flows that are measured by entropy, albeit not a typically causality measure, can effectively show causality properties in our bi-variate system.

#### 2.1.3. Information Flow Measures

The “self-causality” property, or memory of the return series describes the information flow $I_{R\to R}$ and it can be quantified by conditional block entropy as described in Section 2.1.1. We denote $\Delta_{X}\left(k\right)$ as the contribution from memory $x_{t}^{\left(k\right)}$ (see Equation (4)). The larger block size *k*, the larger $\Delta_{X}\left(k\right)$; in our context, it shows the length of the memory available to estimate subsequent price movements and subsequently, return changes.

(4) $$\Delta_{X}\left(k\right)=H_{X}-h_{X}\left(k\right)$$

**Figure 2:**
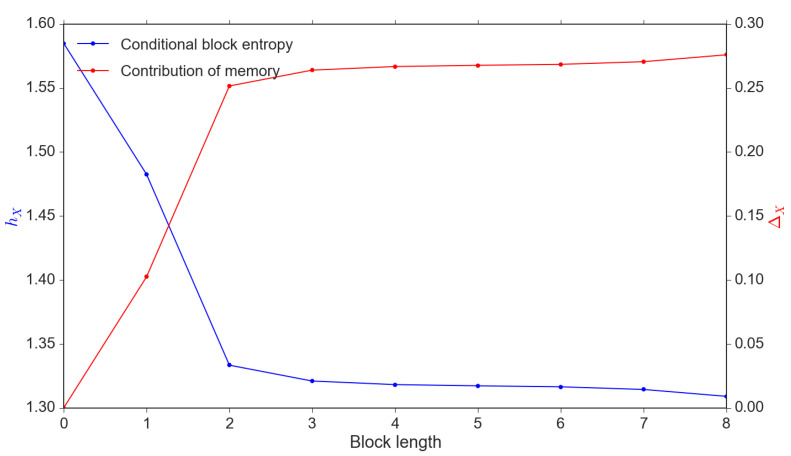
Conditional entropy $h_{X}\left(k\right)$ vs. reduced uncertainty $\Delta_{X}\left(k\right)$. *Note*: These results are calibrated through a simulation sample of 1,000,000 observations.

In Figure 2, we demonstrate that $\Delta_{X}\left(k\right)$ increases until *k* reaches the memory length $k_{X}$. It is clear that the conditional block entropy $h_{X}\left(k\right)$ reduces with the increase in the contribution of the memory $\Delta_{X}\left(k\right)$.

The information flow $I_{S\to R}$ can be regarded as the causal relationship from news sentiment to market return. Hence, we adopt transfer entropy $T_{S\to R}$ to evaluate the amount of information in news that is useful for “forecasting” market returns (see the definition in [36] and Equation (2)). Please note that $T_{S\to R}$ excludes the information transmission from the past market data (returns to returns) and this requires the block size of the return series to be as large as possible in order that the self-causality can be fully extracted. Ideally, the block size of the target information process *k* should be at least equal to the memory length to ensure the robust measure of self-causality. In contrast, the block size of the source, which is the news sentiment in our case, can be determined arbitrarily as it is upon us to decide how far back we would like to trace the influence. These calibration settings of information flow measures are consistent with the understanding from the information discovery literature that historical market data (e.g., prices, returns) is always directly observable and is the most straightforward information to incorporate in trading strategies; hence, in price forecasting, any other information (i.e., news sentiment) must be supplementary information in addition to the full use of market returns.

Another technical issue of to note when applying the entropy measures to evaluate information flows is that they are not directly comparable as their values’ boundaries are different and depend on the sample and parameter selections (see Equation (5)). This has been documented by [12] and we, thereby, follow their approach to linearly map the values to [0.0,1.0] in order to produce comparable values.

(5) $$\left\{\begin{array}{c} 0\leq h_{X}\left(k\right)\leq H_{X}\\ 0\leq T_{Y\to X}\left(k,l\right)\leq h_{X}\left(k\right) \end{array}\right.$$

Finally, we can write down the two information flows as follows:

- market returns → market returns ($I_{R\to R}$)
  (6) $$I_{R\to R}=\frac{\Delta_{R}}{H_{R}}=1-\frac{h_{R}}{H_{R}}$$
- news sentiment → market returns ($I_{S\to R}$)
  (7) $$I_{S\to R}=\frac{T_{S\to R}}{h_{R}}$$

### 2.2. Trading Activities Identification

As discussed before, our focus is to categorize the trading behavior through examining the price discovery based on two types information flows in Equations (6) and (7). The trading behaviors are separated by the information sources that drive the trading and we subsequently get:

- Return-driven trading: Investors are used to follow the market price patterns when making their trading decisions, which is called technical analysis. Such behavior can be identified through self-information flows of market returns. In other words, the memory of market return flow $I_{R\to R}$ is the evidence of return-driven trading according to our model.
- News-driven trading: This often reflects digitization of textual information that allows investors to effectively form beliefs through news and incorporate them into their trading decisions. Such trading strategies pass news sentiment to the market; hence, $I_{S\to R}$ indicates occurrence of news-driven trading.

To sum up, we can form Equation (8) that categorize different types of trading: Positive self information flows in returns define return-driven trading and positive transfer information flows from news sentiment to returns indicate news-driven trading. In the actual modeling, we set the precision of information flows with 4 decimal digits, so that a value lower than 1 basis point will be regarded as 0. Here we concentrate on identifying trading behavior through direct information transmissions at the market level in this bi-variate system and do not go into a further classification of uncommon trading behaviors at micro levels. Hence, we label “other types of trading” relative to the two kinds of trading activities mentioned above in Equation (8).

(8) $$L_{\mathrm{trading}}\left(t\right)=\left\{\begin{array}{cc} \mathrm{Return}\text{-}\mathrm{driven}\;\mathrm{trading}, & I_{R\to R}>0\\ \mathrm{News}\text{-}\mathrm{driven}\;\mathrm{trading}, & I_{S\to R}>0\\ \mathrm{Other}\;\mathrm{types}\;\mathrm{of}\;\mathrm{trading}, & \mathrm{Others} \end{array}\right.$$

### 2.3. Market Information Regime

When viewing the trading activities at the (aggregated) market level, especially when a certain type of trading pattern persists and becomes dominant, it could lead to a market regime. Based on our trading classification, we can count three possible market regimes that are sketched out in Equation (9).

(9) $$L_{\mathrm{regime}}\left(t\right)=\left\{\begin{array}{cc} \mathrm{Return}\text{-}\mathrm{driven}, & I_{R\to R}>0\;\mathrm{and}\;I_{S\to R}=0\\ \mathrm{News}\text{-}\mathrm{driven}, & I_{S\to R}>0\;\mathrm{and}\;I_{R\to R}=0\\ \mathrm{Mixed}, & I_{S\to R}>0\;\mathrm{and}\;I_{R\to R}>0\\ \mathrm{Other}\;\mathrm{types}, & \mathrm{Others} \end{array}\right.$$

(1) The return-driven regime: The market is purely driven by chasing of return patterns. We often obtain stronger return memory in this regime.
(2) The news-driven regime: The market prices moves entirely from responses to news and no self-causality in returns are detected.
(3) The mixed regime: Both return-driven and news-driven trading were identified and they co-exist.
(4) Other types: Neither return-driven nor news-driven trading were detected. The market either react to news and market data too slow to produce significant information flows, or have too few traders using these types of information to form market-level price impacts.

### 2.4. Parameter Settings and Some Calibration Issues

The original data of both market returns and news sentiment are continuous. Instead of fitting the continuous probability density function, we label 3 groups for each of the two time series (see Equation (10)). The labels of market returns capture the price movements of up-trend, no-trend and down-trend; and the labels for news sentiment highlight good, neutral and bad financial/business news. The reasons for using discrete probabilities are twofold. First, estimating continuous probability density functions is both data-intensive and computing-intensive. Second, investors usually make decisions based on their optimistic or pessimistic prospect, for example forecasting of bull and bear market, or chasing positive returns.

(10) $$L\left(t\right)=\left\{\begin{array}{cc} -1, & x(t)<\mu -d\\ 0, & \mu -d\leq x(t)\leq \mu +d\\ 1, & x(t)>\mu +d \end{array}\right.$$

When labeling the returns or sentiment, we refer to the data partition approach in [12] that finds a threshold *d* to group the 3 states into approximately the same probability (i.e., $p\left(L=-1\right)\approx \;p\left(L=0\right)\;\approx p\left(L=1\right)\approx \frac{1}{3}$). The literature often adopts a so-called “optimal alphabet partition problem” for data discretization. However, according to [12], equal probability partition fits better to our problem considering the advantages of “neutralising undesirable effects due to very in-homogeneous histograms and ignoring the trivial information gain obtained by just observing marginal distributions.” As an important technique for information disclosure, equal probability partitioning has been well explored in the literature (see [37,38,39]). The implementation of this partitioning method is introduced in Appendix C.

**Figure 3:**
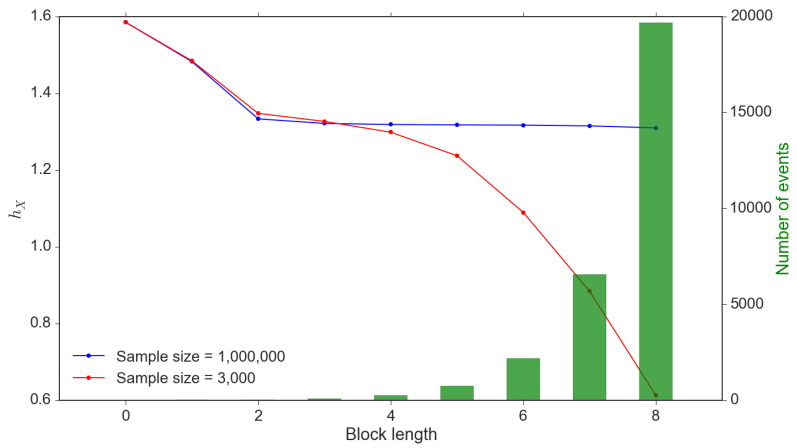
Small sample bias of $h_{I}\left(k\right)$. *Note:* This is an interpretation of systematically undervaluing conditional entropy due to small sample size. This calibration issue exists in transfer entropy as well. These values are calibrated through a simulation sample of 1,000,000 and 3000 observations.

The accuracy of entropy calibration relies highly on the sample size. Theoretically, the sample size should be much larger than the number of events in the probability space to avoid systematically undervaluing entropy. However, this criterion may not be satisfied due to the exponentially increasing number of events with increasing block sizes. We demonstrate this in Figure 3 and it is clear that a small sample size leads to significant undervaluation, especially poor ability to uncover the number of events within the probability space. In contrast, a much larger sample size would provide stable estimations; however, is unrealistic to obtain that many data observations.

To address this issue, we apply the method introduced by [40] to estimate entropy through fitting a monotonically decreasing frequency function. The rationale of this method is that most statistical properties, including entropy, are purely a matter of probability density so that the order of events can be ignored. The key of this method is to design a function that can be turned to different shapes but not too complex. Reference [40] confirmed the best results in their experiments can be presented as follows:

(11) $$p\left(k\right)=\left\{\begin{array}{cc} \alpha {\left(k-\epsilon \right)}^{-\frac{1}{3}}, & 1\leq k\leq \beta \\ \phi k^{-\delta }, & \beta \leq k\leq \gamma \\ 0, & k>\gamma \end{array}\right.$$

This estimation approach is applied on both conditional block entropy and transfer entropy. To fully capture the strength of self information flow, we need to solve the optimization problem of the memory length $k_{X}$ (see Equation (12)).

(12) $$k_{X}=\arg\max_{k}\Delta_{X}\left(k\right)$$

**Figure 4:**
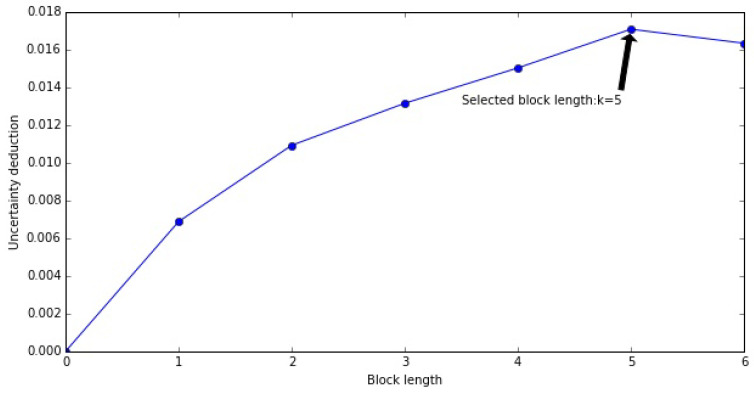
Annotation of memory length optimization and selection

In practice, the cut-off memory length may not be as clear as the simulated samples in Figure 3 due to limited sample size or data noise so that such strict selection criteria may not be applicable. We set a threshold c=10% which the first *k* that satisfies Equation (13) can be determined for the memory length of *X* (see Figure 4 showing the optimal block length of memory).

(13) $$\frac{\Delta_{X}\left(k\right)-\Delta_{X}\left(k-1\right)}{\Delta_{X}\left(k-1\right)}<c$$

As indicated above, in transfer entropy $T_{Y\to X}\left(k,l\right)$, the block size of *X* should be the optimized value $k=k_{X}$. In addition, we only test one period cross-sectional influence so that the block size of *Y* is always fixed to l=1.

In this study, all information entropy measures are calculated through a rolling window of 1-year: the window rolls on daily basis and the window length is one year that gives sufficient observations to capture any major statistical relations in the market even under the extreme market conditions such as a crisis. Within each moving window, we have around 3300 time series observations at a 30-min data frequency (detailed data descriptions are in Section 3). We firstly compute the daily information flows then average them to a weekly frequency. Intuitively, the daily information flow should not change dramatically, while some noise in calibration may be inevitable. The reason that we roll the window on a daily basis is to reduce calibration bias in the weekly proxies.

## 3. Data

The market and financial news sentiment data for this research are obtained from Thomson Reuters Tick History (TRTH) and Thomson Reuters News Analytics (TRNA) respectively. The dataset is in 30-min frequency from 1 January 2003 to 31 December 2014, excluding non-trading hours.

### 3.1. Financial Market Data

Stock market indices are proxies of equity market movements. In this study, we use S & P 500 (.SPX) index prices to represent the U.S. stock market. This index involves large-cap equities which usually have high trading liquidity so that the price movements are sensitive to traders’ responses to real-time information. In other words, information flows can be most accurately measured without being affected by transaction issues. We collect 30-min intraday prices of the market index from TRTH database.

### 3.2. News Sentiment Data

In this research, we select TRNA data to compute news sentiment for two reasons. First, Thomson Reuters is a top financial data vendor, providing complete and reliable news data feeds. Second, TRNA is a professional news sentiment database that has been adopted by previous studies [41]. TRNA adopts natural language processing techniques to read and score news articles in real time (TRNA is a component of Thomson Reuters Machine Readable News. Detailed introductions can be found in https://developers.refinitiv.com/sites/default/files/ThomsonReutersMRNElektronDataModelsv210_2.pdf). In the TRNA database, sentiment is measured as positive, negative and neutral probabilities which allow us to customize the formula for our sentiment score. In addition, it provides a separate record for each company mentioned in every single piece of news articles to show relevance of the news to individual stocks. The relevance score suggests whether a company plays a main role in the news. It is common that a news article has strong sentiment while weak relevance to some stocks mentioned in it. We use the relevance score to tune the sentiment to a lower level in this case.

The metadata fields we used for sentiment calibration in this paper are listed below.

- datetime: The date and time of a news article.
- ric: Reuters Instrument Code (RIC) of a stock for which the sentiment scores apply.
- pos, obj, neg: Positive, neutral, and negative sentiment probabilities (i.e., pos+obj+neg=1).
- relevance: A real-valued number between 0 and 1 indicating the relevance of a piece of news to a stock. One news article may refer to multiple stocks. A stock with more mentions will be assigned a higher relevance.

To evaluate the sentiment score of each record (i.e., one score per news per stock), we calculate the expectation of sentiment probabilities adjusted by relevance value (see Equation (14)).

(14) sentiment=relevance×(pos−neg)×(1−obj)

As we use the .SPX to represent the U.S. market, we track the components of this index over time and only count the news related to these stocks. Changes of the .SPX index constituents are obtained from The Compustat. Then we define 30-min news sentiment as the average sentiment of all records published within the time interval. The news released in non-trading hours are counted into the first 30-min of the following trading day.

### 3.3. Stationarity Test

We apply the augmented Dickey–Fuller (ADF) test on the price data, log-returns, and news sentiment. The null hypothesis is that there is a unit root. The results in Table 1 show that for returns and sentiment, the null hypothesis is rejected at a strong 99.9% confidence level. In contrast, the price series is apparently non-stationary as expected. These results confirm that our model setting of using returns and sentiment for information flow computation is valid.

## 4. Results

We highlight two types of information flows as proxies of trading behaviors in Section 2: $I_{R\to R}$ for return-driven trading; and $I_{S\to R}$ for sentiment-driven trading. We present key findings of these information flows in this section.

**Figure 5:**
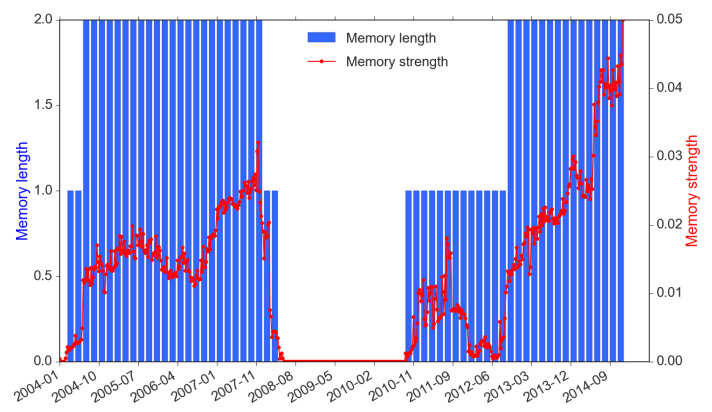
Self information flow (memory) of market returns (2004–2014).

$I_{R\to R}$ is a self-causality information flow, which can be regarded as the “memory” of the return time series. The memory length and strength are equivalent to the block size and the standardized entropy value. From the time series perspective, return memory is associated with a price trending or reversal pattern, and the strength of memory indicates the scale of the dominance of such patterns over the price movements. According to Figure 5, the memory strength of market returns clusters into three time periods: pre-crisis (before 2008), crisis (2008–2011, covering both 2008 liquidity crisis and EuroDebt crisis) and post-crisis (after 2013). As self information flow $I_{R\to R}$ is the return-driven trading proxy, we observe that most return-driven trading responses to market based on the past two 30-min periods (1 hour) in the pre- and post-crisis. We also observe that stronger information flows coincide with strong memory length (e.g., the strongest $I_{R\to R}$ has reached 0.05 in late 2014).

Recall that we consider a 1-year rolling window to incorporate sufficient data to obtain the optimal memory length reflecting the impact on the market. In this case, the information flow of each point at time *t* actually represents an accumulative effect of the past year prior to time *t*. Therefore, the self-causality of market returns, which appears in a cyclic pattern, is closely associated with events such as financial crises that are often triggered by persistent pre-crisis activities and spread with contagions after the outbreak of crises. During the crisis period, however, there are a few interesting and unique findings. First, throughout the 2008 crisis and early period of the EuroDebt crisis (August 2008 to November 2011), both the memory length and strength have stayed at zero. We think it is because both the 2008 liquidity crisis and 2011 EuroDebt crisis have caused fundamental structural changes to the market and led to investors’ completely different ways to respond after being shocked during this period. This period began just before the Lehman’s official filing of bankruptcy and endured for some time even until the occurrence of the EuroDebt crisis.

No one is sure how long exactly that the 2008 crisis may have affected the market; but inevitably, the Eurozone sovereign debt that started in early 2010 could only make the market more stressed. This explains why return memory suddenly dropped and remained absolutely static at the zero position, which also indicates traders stayed away from return-driven trading activities. However, differing from the 2008 crisis, the European Central Bank (ECB), together with the European Financial Stability Facility (EFSF) and European Stability Mechanism (ESM), had swiftly taken a much more systematic approach to solve the EuroDebt crisis and the market started to calm down subsequently (The ECB, on 6 September 2012, extended its approach by providing free unlimited support for affected countries through the EFSF/ESM’s state bailout/precautionary program). Therefore, the information flow of market returns, in terms of both memory length and strength, picked up from late 2010. Another reason the entropy memory length and strength are partially affected during the EuroDebt crisis could be that the cross-market spillover effects were not as strong or long-lasting as the 2008 crisis’ direct impact on the US market. When the market calmed down even further since 2013, the memory length came back to the pre-crisis level of 1 h and the strength outweighed the maximum of pre-crisis level (0.05 vs. 0.03).

**Figure 6:**
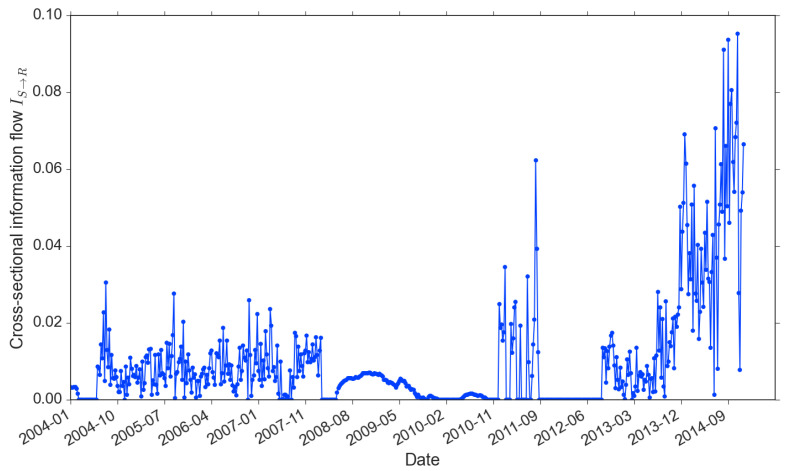
Information flow from news sentiment to market return (2004–2014).

The other information flow $I_{S\to R}$ is the proxy for news-driven trading. In Figure 6, we observe that, similar to the return-driven trading, the news-driven trading is persistently involved in the market. The only exception is from late 2011 to early 2013, right after the EuroDebt crisis. It is also the period that the market started to pick up after a few years of downturn. As return updates faster than news, the absence of news-driven trading reflects the adaptiveness of investors. They tend to firstly response to the more timely and better organized information. We also observe that the information flow $I_{S\to R}$ existed during the 2008 crisis: in contrast with the $I_{R\to R}$, which stayed zero. It confirms our previous argument that investors changed their way of trading after the bubble burst, from responding to price patterns to decisions on beliefs of news. Similar to the memory of return, $I_{S\to R}$ also increased sharply with the market recovering from 2013.

**Figure 7:**
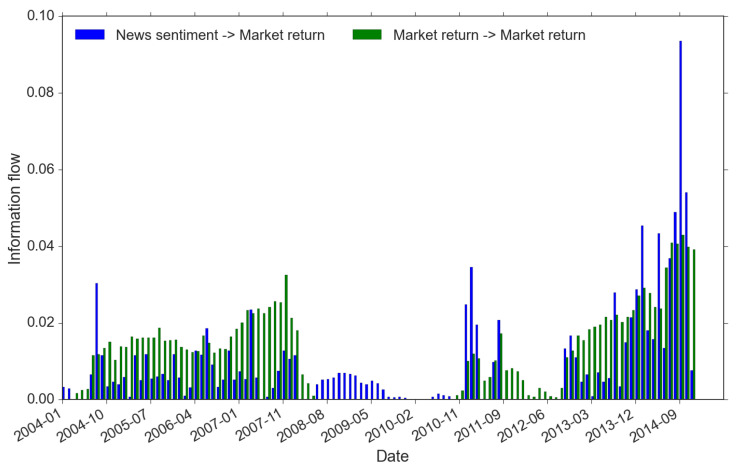
Market regimes.

We identify market regimes using the criteria described in Equation (9) and these regimes are formed through different trading activities. We focus on explaining three market regimes, namely return-driven, news-driven and the mixed regimes (see Equation (9)). Technically, these regimes are recognized if the information flow exceeds 1 basis point, the precision we set for all information flow measures. Because our information flow is calculated with a 1-year rolling window, it actually reveals insights of traders’ behavior in the past. This is highly important in that the trading behaviors detected in the market actually reflects the accumulative effects of historical trading activities, rather than just the contemporary trading impact on the market. For instance, Figure 7 suggests that, in the first half of 2010, the market should be relatively slow moving because in almost the first 10 months in 2010 it appears to lack signs of both news-driven and return-driven trading (we define it as “other types” in Equation (9)). In fact there is no sign of market-driven as far back as early-to-mid 2009. Nobody in the market would disagree with this finding as this is not long after the official filing of the bankruptcy of Lehman in August 2008. The market has already been severely shaken, market participants are extremely cautious, and regulators are highly alerted.

From Figure 7, we summarize below the key results regarding market regimes:

- There are two periods within which the market regime is driven by a single type of trading activity: (1) from the Q3 of 2008 to the Q4 of 2010, the single source of market-wide trading is news sentiment (blue bars only); while (2) from the Q4 of 2011 to the Q3 of 2012, the return memory clustering indicates return-driven activities that drive the market movements (green bars only). Before and after the news-driven regime (period 1 here), we spot a swift switch from returns to sentiment. However, for the return-driven regime (period 2 here), instead, it is more of the fact that news influence disappears from a mixed-regime. These signs are important because they could be highly indicative. They show that news sentiment always requires longer time to form compared to the belief towards some fast updating changes in the market (e.g., reflected in returns).
- During the rest of the time, price movements are caused by mixed types of trading. In addition, the mixed regime demonstrates strong features associated with the market crisis timeline. In the pre-crisis period (before 2008), although there exists trading of both returns and news, often return-driven trading overpowers the news-driven (apart from one exceptional spike of news event around October 2010); while in the post-crisis period (after 2013), the dominance more often resides in the power of news-driven trading, moreover, at a much higher level than the return-driven. This finding is of great interest to us because it provides strong evidence of the change in the market regimes’ dynamics before and after the double crisis period. Furthermore, the imbalance between their dominants within the mixed regime has changed dramatically and more frequently in the post-crises years. We see a few flash spikes in news-driven trading, while there was only one spike showing clear imbalance around October 2004 during the pre-crisis period. All these suggest that the complexity of the market may have increased after the crises with the growth of modern technology and big data [42].

These observations highlight an adaptive pattern of investors’ trading behaviors, which naturally imply the dynamics of underlying information discovery: before the 2008 financial crisis, the global economy enjoyed a few years of boom and investors were confident and optimistic about the bull market and kept chasing prices [43]. During the same time period, digitization of textual information allowed business news to be widely adopted in investment decisions. Access to innovative information brings new opportunities for excess returns. This explains why the news-driven trading was actively involved, but not primarily dominant, in the financial market during the pre-crisis period.

In the double crisis periods, trading activities were mainly led by news. This is because, under the extreme market condition, the underlying price generating process was apparently far from what could be interpreted by widely adopted financial models. The market has been gloomy and the general confidence of price movements are destroyed as market participants are confused. Therefore, we observe the trading dominated by news and the investors were very “quiet” toward market return information. There was a short time (around March to June, 2012) that no particular types of market activities or regimes could be identified. Most investors were managing their investment passively and panic about unforeseen changes.

Finally, investors cannot obtain full information transparency. This argument links the market efficiency problem to the information competition among investors. To be more specific, when the majority of investors hold back in the information competition, gaps of price discovery start to emerge. Therefore, after a few years of weak or no trading using news and returns, an even stronger information flow shows up from 2013. In addition, as we summarized before, the market has become much more complex, even in the formation of news sentiment. At the same time, with the rapid growth in new financial technology and data science, the complexity of the financial system has been further enhanced through complex trading techniques, for instance, ultra-high frequency trading.

## 5. Conclusions

This study is innovative in applying information entropy to identify trading activities. In our model, the financial market is considered as a bivariate system of news sentiment and market return. Entropy measures the causality relationships of these two time series to indicate the information flowing in this system. We argue that information transmission in this system represents two types of trading behaviors: return-driven trading that can be identified through self-causality of market return, and news-driven trading which is revealed by the cross-sectional information flow from news sentiment to market return. From the economic perspective, this study applies 11 years of news sentiment and market data to show the evolution of financial market regimes in terms of adaptive trading activities. The proposed method can be expanded to study more comprehensive types of information that lead to trading decisions.

There are some limitations in this study. We recognize there are different approaches to measuring news sentiment [19,20]. We use a commercially available one from Thomson Reuters. We recognize that there is no universally agreed news sentiment measure, nor a universally adopted method to map textual information to investor beliefs. The accuracy of such a measure may affect the “level of sentiment information used in trading”. Nevertheless, all different investor sentiment measures have been approved correlated, and there is always a need for better quality and reproducibility of the proposed measures [44,45]. Although such variance may not affect the main findings we document in this study, the differences in effect and accuracy should be examined. Moreover, this study only focuses on news-driven and return-driven trading behaviors. We are silent about other types of trading activities, if any, and consequently more market regime delineations. We recommend future studies to discover other behaviors using or extending the proposed methodology, and examine their effects on the market price formation.

## Figures and Tables

**Table 1:** ADF test results.

	t-Statistic	*p*-Value
Price level	−0.631	0.864
Log-return	−27.092	0.000
News sentiment	−10.901	0.000

Null hypothesis: there is a unit root. Alternative hypothesis: the time series is stationary. Regression model includes a constant and no trend.

**Figure A1:**
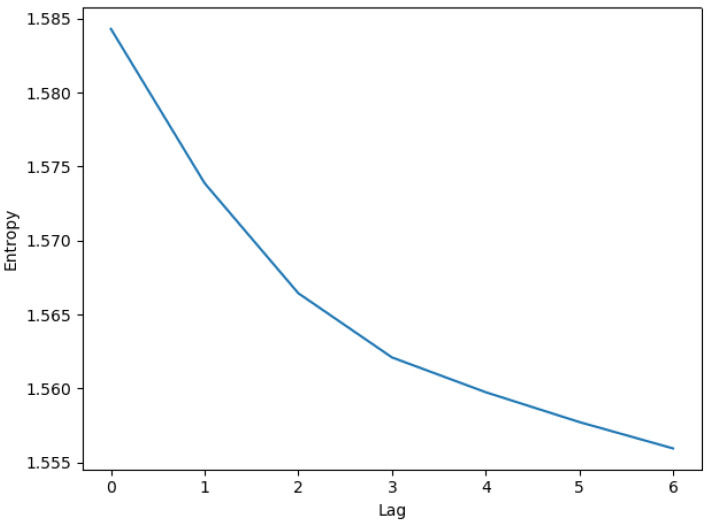
Decreasing entropy with increasing information memory.

## Appendix A

This appendix presents proofs of theorems of well-known facts related to entropy measures.

**Theorem** **A1.**
*If X is a sequence of i.i.d. random variables, then there is no self information flow within the series X i.e., the conditional block entropy shall be equal to the Shannon entropy.*

**Proof.** For an i.i.d. sequence *X*, we have

(A1) $$\begin{array}{c} p\left(x_{t+1}|x_{t}^{\left(k\right)}\right)=p\left(x_{t+1}\right) \end{array}$$

(A2) $$\begin{array}{c} \;\mathrm{and}\;p\left(x_{t+1},x_{t}^{\left(k\right)}\right)=p\left(x_{t}^{\left(k\right)}\right)p\left(x_{t+1}|x_{t}^{\left(k\right)}\right)=p\left(x_{t}^{\left(k\right)}\right)p\left(x_{t+1}\right) \end{array}$$

Then from Equation (1)

(A3) $$\begin{array}{cc} h_{X}\left(k\right) & =-\sum p\left(x_{t+1},x_{t}^{\left(k\right)}\right)\log_{2}p\left(x_{t+1}|x_{t}^{\left(k\right)}\right)\\ \; & =\sum p\left(x_{t}^{\left(k\right)}\right)\left\{-\sum p\left(x_{t+1}\right)\log_{2}p\left(x_{t+1}\right)\right)\}\\ \; & =\sum p\left(x_{t}^{\left(k\right)}\right)H_{X}\\ \; & =H_{X} \end{array}$$

Since $\sum p(X_{t}^{\left(k\right)})=1$. □

**Theorem** **A2.**
*For two independent series X and Y, the transfer entropy between them will be zero (i.e., no causal relationships between X and Y).*

**Proof.** For the two series *X*, *Y*, the transfer entropy satisfies Equation (2). If the two series are independent, we have $p\left(x_{t+1}|x_{t}^{\left(k\right)},y_{t}^{\left(l\right)}\right)=p\left(x_{t+1}|x_{t}^{\left(k\right)}\right)$. Then for all possible series values the logarithmic term in the above expression becomes $log_{2}\left(1\right)=0$. So $T_{Y\to X}=0$ for any positive integers *k* and *l*. Similarly, $T_{X\to Y}=0$ as well. □

**Theorem** **A3.**
*Granger causality and transfer entropy are equivalent if all variables involved are distributed as multivariate normal distributions.*

**Proof.** This is a more succinct proof of a result of [27]. For any random vector *Z* with probability density f(Z) the entropy is defined as

(A4) $$\begin{array}{c} H(Z)=-\int f(z)\ln f(z)dz=-E[\ln f(Z)]. \end{array}$$

Please note that we are using ”Natural” logarithms rather than base 2 logs that are common in information theory. If *Z* has multi-Normal distribution Z∼MN(μ,Σ(Z)) the probability density is

(A5) $$\begin{array}{cc} f(z)= & {\left(2\pi \right)}^{-\frac{1}{2}d_{Z}}{\left|\Sigma \left(Z\right)\right|}^{-\frac{1}{2}}\\ & \exp\left\{-\frac{1}{2}{\left(z-\mu \right)}^{\prime }\Sigma {\left(Z\right)}^{-1}\left(z-\mu \right)\right\}, \end{array}$$

where $d_{Z}$ is the dimension of Z. Then

(A6) $$\begin{array}{cc} H(Z) & =\frac{1}{2}d_{Z}\ln\left(2\pi \right)+\frac{1}{2}\ln\left|\Sigma \left(Z\right)\right|\\ \; & +E[\frac{1}{2}{\left(Z-\mu \right)}^{\prime }\Sigma {\left(Z\right)}^{-1}\left(Z-\mu \right)]. \end{array}$$

However, the quadratic form in the final term has a chi-squared distribution with $d_{Z}$ degrees of freedom, and so has expectation $d_{Z}$. Therefore

(A7) $$\begin{array}{cc} H(Z) & =\frac{1}{2}\ln\left|\Sigma \left(Z\right)\right|+\frac{1}{2}d_{Z}ln\left(2\pi \right)+\frac{1}{2}d_{Z}\\ & =\frac{1}{2}\ln\left|\Sigma \left(Z\right)\right|+\frac{1}{2}d_{Z}ln\left(2\pi e\right). \end{array}$$

Now let $Z=\left(\begin{array}{c} X\\ W \end{array}\right)$, then Equation (A5) can be written as

(A8) f(z)=f(w)f(x|w),

where f(w), similar to Equation (A5),

(A9) $$\begin{array}{cc} f(w)= & {\left(2\pi \right)}^{-\frac{1}{2}d_{W}}{\left|\Sigma \left(W\right)\right|}^{-\frac{1}{2}}\\ & \exp\left\{-\frac{1}{2}{\left(w-\mu_{W}\right)}^{\prime }\Sigma {\left(W\right)}^{-1}\left(w-\mu_{W}\right)\right\}, \end{array}$$

The conditional density is

(A10) $$\begin{array}{cc} f(x|w)= & {\left(2\pi \right)}^{-\frac{1}{2}d_{X}}{\left|\Sigma \left(X|W\right)\right|}^{-\frac{1}{2}}\\ & \exp\left\{-\frac{1}{2}{\left(x-\mu_{X|W}\right)}^{\prime }\Sigma {\left(X|W\right)}^{-1}\left(x-\mu_{X|W}\right)\right\}, \end{array}$$

where the conditional dispersion matrix is

(A11) $$\begin{array}{c} \Sigma \left(X|W\right)=\Sigma \left(X\right)-\Sigma \left(X,W\right)\Sigma {\left(W\right)}^{-1}\Sigma \left(W,X\right) \end{array}$$

with

(A12) $$\begin{array}{c} \Sigma \left(Z\right)=\Sigma \left(\begin{array}{c} X\\ W \end{array}\right)=\left(\begin{array}{cc} \Sigma (X) & \Sigma (X,W)\\ \Sigma (W,X) & \Sigma (W) \end{array}\right). \end{array}$$

Please note that from Equations (A5) and (A8) to (A10)

(A13) $$\begin{array}{c} |\Sigma (Z)|=|\Sigma (W)||\Sigma (X|W)|. \end{array}$$

Let $x_{t+1},x_{l}^{\left(k\right)},y_{t}^{\left(l\right)}$ have a multivariate Normal distribution. Then transfer entropy is

(A14) $$\begin{array}{cc} T_{Y\to X}\left(k,l\right) & =H\left(x_{t+1}|x_{t}^{\left(k\right)}\right)-H\left(x_{t+1}|x_{t}^{\left(k\right)},y_{t}^{\left(l\right)}\right)\\ \; & =\frac{1}{2}\ln\left|\Sigma \left(x_{t+1}|x_{t}^{\left(k\right)}\right)\right|+\frac{1}{2}\ln\left(2\pi e\right)\\ \; & -\frac{1}{2}\ln\left|\Sigma \left(x_{t+1}|x_{t}^{\left(k\right)},y_{t}^{\left(l\right)}\right)\right|-\frac{1}{2}\ln\left(2\pi e\right)\\ \; & =\frac{1}{2}\ln\left\{\frac{\Sigma (x_{t+1}|x_{t}^{\left(k\right)})}{\Sigma (x_{t+1}|x_{t}^{\left(k\right)},y_{t}^{\left(l\right)})}\right\} \end{array}$$

The argument of the logarithm is just the ratio of the variance of $x_{t+1}$ conditional on $x_{t}^{\left(k\right)}$ and the variance of $x_{t+1}$ conditional on both $x_{t}^{\left(k\right)}$ and $y_{t}^{\left(l\right)}$. As we are dealing with multivariate Normal, these are calculated by appropriate forms of Equation (A11), which is a standard result for linear regression (whether or not distributions are Normal). This is therefore exactly the criterion that is used to determine whether *Y* Granger causes *X*, and so Granger causality and transfer entropy are equivalent if all variables involved are distributed as multivariate Normal. □

## Appendix B

We apply the vector autoregression (VAR) and Granger causality tests on the entire dataset to build a linear model of our bi-variate system. This model is then compared with a model using entropy measures. We set the maximum lags of 6 for both groups of models, then choose the optimal lag and memory length using information criteria or the method proposed in this paper respectively.

The optimal lag selected for the VAR model according to the Akaike information criterion (AIC) is 6. As our focus of trading activity identification only considers the lagged impacts of return and sentiment to the return series, we only tabulate the equation of return in the VAR model (see Table A1).

Using a 95% confidence level, we find the lag-2 and lag-6 return coefficients and the lag-4 sentiment are significant. The VAR model considers information of different lags separately. Hence, we would see “jumps” of lags. In contrast, entropy measures take information filtration to identify lagged impacts. For example, the conditional block entropy of lag-*n* indicates how much uncertainty of the current data is explained by information from *n*-period ago to right before the present. Apparently, the amount of information increases with lags so that reduces entropy gradually (see Figure A1). As traders would not intentionally skip certain time periods while gathering information for trading, the idea of entropy matches better with the real trading activities.

**Table A1:** VAR model results.

	Coefficient	T-Stat	*P*-Value
Const.	0.0000	0.235	0.814
Lag-1 return	0.0018	0.352	0.725
Lag-1 sentiment	0.0003	1.086	0.278
**Lag-2 return**	0.0129	2.546	0.011
Lag-2 sentiment	−0.0001	−0.227	0.821
Lag-3 return	0.0079	1.574	0.116
Lag-3 sentiment	−0.0002	−0.900	0.368
Lag-4 return	0.0014	0.278	0.781
**Lag-4 sentiment**	0.0006	2.320	0.020
Lag-5 return	−0.0025	−0.489	0.625
Lag-5 sentiment	−0.0003	−1.057	0.290
**Lag-6 return**	−0.0208	−4.121	0.000
Lag-6 sentiment	0.0001	0.290	0.771

According to the memory length selection method introduced in this paper, we get the optimal 3-period memory for return data, which is shorter than the selection of 6 lags for the VAR model. As explained above, this is because the VAR model tends to omit the impacts of some insignificant lags due to the linearity assumption and as a result missed some information. Using this memory length, we get the information flow from lag-1 sentiment to return is 10 basis point, which is much lower than the 1.55% information flow from return to itself. This is consistent with the linear model results above that coefficients of lagged sentiment is much smaller than those of lagged returns. Furthermore, despite the fact that traders are actively tracking news updates in trading, the Granger causality test fails to identify the impacts from sentiment to return (see Table A2).

**Table A2:** Granger causality test *p*-values.

	Lag-1	Lag-2	Lag-3	Lag-4	Lag-5	Lag-6
Sentiment → Return	0.2235	0.4793	0.6106	0.1492	0.1654	0.2340
Return → Sentiment	0.2906	0.4838	0.0861	0.1299	0.0028	0.0047

## Appendix C

We discritize both return and sentiment into 3 partitions, i.e.

$$L\left(t\right)=\left\{\begin{array}{cc} -1, & x(t)<\mu -d\\ 0, & \mu -d\leq x(t)\leq \mu +d\\ 1, & x(t)>\mu +d \end{array}\right..$$

in which μ is the mean of the data and *d* is the threshold for partition.

The equal probability means $p\left(L=-1\right)=p\left(L=0\right)=p\left(L=1\right)=\frac{1}{3}$. However, if the data is asymmetric, the results would be diverged. Hence, the problem is to minimize the divergence of distribution. We use the Kullback-Leibler divergence

$$D_{\mathrm{KL}}\left(P\|Q\right)=\sum_{x\in X}P\left(x\right)\log\left(\frac{P(x)}{Q(x)}\right).$$

in which P(x) is our partition results and Q(x) is the equal probability density of that has the same probability $\frac{1}{3}$ for each partition. The optimization problem is to find the *d* that minimize the distance $D_{\mathrm{KL}}\left(P\|Q\right)$.

In fact, both return and sentiment data are almost symmetrical. We set the initial value of *d* as the $\frac{2}{3}$ quantile of the data so that the optimization converges fast. The results are in Table A3.

**Table A3:** Equal probability partition results.

	μ	*d*	$D_{\mathrm{KL}}\left(P\|Q\right)$
Return	0.0	0.000631	0.00046
Sentiment	0.05	0.029146	0.0025
